# The Role of Eicosanoids in Gynecological Malignancies

**DOI:** 10.3389/fphar.2020.01233

**Published:** 2020-08-26

**Authors:** Paige G. Smith, Dana Roque, Mc Millan Ching, Amy Fulton, Gautam Rao, Jocelyn C. Reader

**Affiliations:** ^1^ Department of Obstetrics, Gynecology and Reproductive Medicine, University of Maryland School of Medicine, Baltimore, MD, United States; ^2^ Marlene and Stewart Greenebaum Comprehensive Cancer Center, Baltimore, MD, United States; ^3^ Cellular and Molecular Medicine, Johns Hopkins University School of Medicine, Baltimore, MD, United States; ^4^ Department of Pathology, University of Maryland School of Medicine, Baltimore, MD, United States; ^5^ Baltimore Veterans Administration Medical Center, Baltimore, MD, United States

**Keywords:** gynecological cancers, eicosanoid, lipoxygenase, epoxygenase, cyclooxygenase

## Abstract

Eicosanoids, bio-active lipid molecules, evoke a multitude of biological effects that directly affect cancer cells and indirectly affect tumor microenvironment. An emerging role has been shown for eicosanoids in the pathogenesis of gynecological malignancies which include cancers of the vulva, vagina, cervix, uterine, and ovary. Eicosanoid biosynthesis pathways start at the metabolism of phospholipids by phospholipase A2 then proceeding to one of three pathways: the cyclooxygenase (COX), lipoxygenase (LOX), or P450 epoxygenase pathways. The most studied eicosanoid pathways include COX and LOX; however, more evidence is appearing to support further study of the P450 epoxygenase pathway in gynecologic cancers. In this review, we present the current knowledge of the role of COX, LOX and P450 pathways in the pathogenesis of gynecologic malignancies. Vulvar and vaginal cancer, the rarest subtypes, there is association of COX-2 expression with poor disease specific survival in vulvar cancer and, in vaginal cancer, COX-2 expression has been found to play a role in mucosal inflammation leading to disease susceptibility and transmission. Cervical cancer is associated with COX-2 levels 7.4 times higher than in healthy tissues. Additionally, HPV elevates COX-2 levels through the EGFR pathway and HIV promotes elevated COX-2 levels in cervical tissue as well as increases PGE_2_ levels eliciting inflammation and progression of cancer. Evidence supports significant roles for both the LOX and COX pathways in uterine cancer. In endometrial cancer, there is increased expression of 5-LOX which is associated with adverse outcomes. Prostanoids in the COX pathway PGE_2_ and PGF_2α_ have been shown to play a significant role in uterine cancer including alteration of proliferation, adhesion, migration, invasion, angiogenesis, and the inflammatory microenvironment. The most studied gynecological malignancy in regard to the potential role of eicosanoids in tumorigenesis is ovarian cancer in which all three pathways have shown to be associated or play a role in ovarian tumorigenesis directly on the tumor cell or through modulation of the tumor microenvironment. By identifying the gaps in knowledge, additional pathways and targets could be identified in order to obtain a better understanding of eicosanoid signaling in gynecological malignancies and identify potential new therapeutic approaches.

## Introduction 

Eicosanoids, bio-active lipid molecules, elicit a wide range of biological effects that plays a role in physiological and pathophysiological conditions (Harizi et al., 2008; Buczynski et al., 2009; Chhonker et al., 2018). Eicosanoid driven processes have been shown to have increasing importance in the development, progression and metastasis of gynecological malignancies. Gynecological malignancies are cancers that initiate in the reproductive organs of women that include vulvar, vaginal, cervical, uterine, and ovarian cancers. The eicosanoid pathway in cancer has been reviewed in detail (Panigrahy et al., 2010; Wang and DuBois, 2010; Gomes et al., 2018; Umamaheswaran et al., 2018); briefly, arachidonic acid (AA) is liberated from membrane phospholipids by phospholipase A2 (PLA2) and metabolized by one of three pathways cyclooxygenase (COX), lipoxygenase (LOX) and P450 epoxygenase which then produce a wide range of prostanoids, leukotrienes, epoxyeicosatrienoic acids (EETs), and hydroxyeicosatetraenoic acids (HETEs) (Wang and DuBois, 2010; Gomes et al., 2018). The most prominent eicosanoids to be identified to play a role in cancer are those involved in the COX and LOX pathways (Jones et al., 2019); however, products of the cytochrome P450 epoxygenase pathway are shown to play an emerging role in angiogenesis, inflammation and cancer [as reviewed by (Panigrahy et al., 2010)]. In gynecological malignancies, eicosanoids can act directly on the cancer cells, indirectly in the tumor microenvironment and, in many cases, at the confluence of infectious diseases such as HIV and HPV, inflammation and carcinogenesis (De Nola et al., 2019). This review will highlight the role of eicosanoids in the development and progression of gynecological malignancies.

**Figure 1:**
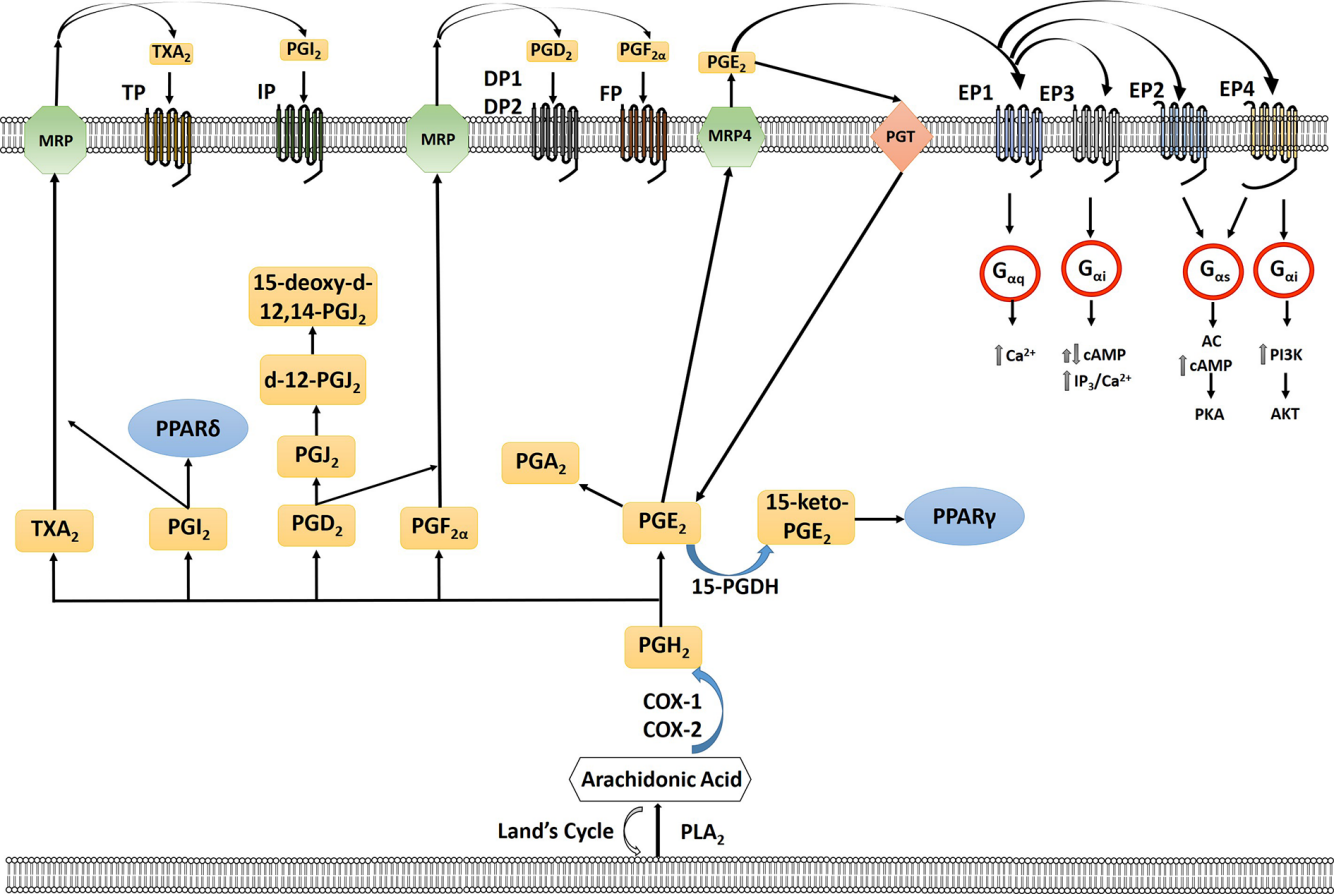
Arachidonic acid (AA) is liberated from the plasma membrane *via* phospholipase A2 (PLA2). AA can be recycled by the Lands cycle, which is a reacylation/deacylation cycle, that serves to keep the concentration of free AA at a low level. AA is converted by cyclooxygenase 1 or 2 (COX-1/COX-2) to PGH_2_ which is then converted to PGE_2_, PGF_2α_, PGD_2_, PGI_2_, or TXA_2_ by prostaglandin specific synthases. PGE_2_ is exported out of the cell by multidrug resistance-associated protein four (MRP4) where it can bind to its receptors, the E series of prostaglandin receptors on the plasma membrane, EP1-4. Each of the G-protein-coupled- receptors signal through a different intracellular pathway: EP1 leads to elevation of intracellular calcium through Gαq, EP3, which exists in multiple isoforms, can lead to different responses with the majority acting to inhibit cAMP through Gαi as well as an increase in IP3/intracellular calcium; EP2 and EP4 cause stimulation of cyclic AMP (cAMP) production and protein kinase A (PKA) by sequential activation of Gαs and adenylate cyclase (AC); EP4 can also activate phosphoinositide-3-kinase (PI3K) through Gαi. PGE_2_ is imported back into the cell through prostaglandin transporter (PGT) where it can either be re-exported or inactivated by 15-hydroxyprostaglandin dehydrogenase (15-PGDH) to 15-keto-PGE_2_. 15-keto-PGE_2_ can signal through PPARγ. PGE_2_ can be converted to PGA_2_ through a dehydration reaction. PGD_2_ through a series of dehydrogenation reactions creates PGJ_2_, delta-12-prostaglandin J2 (d-12-PGJ_2_) and 15-deoxy-delta12,14-prostaglandin J2 (15-d-PGJ_2_). PGI_2_ can either signal through PPARδ intracellularly or exported *via* multidrug resistance protein (MRP) to signal through the IP receptor. TXA_2_ is exported out of the cell *via* MRP to signal through the TP receptor. PGF_2α_ is exported out of the cell *via* MRP where it can signal *via* the FP receptor on the cell surface.

**Figure 2:**
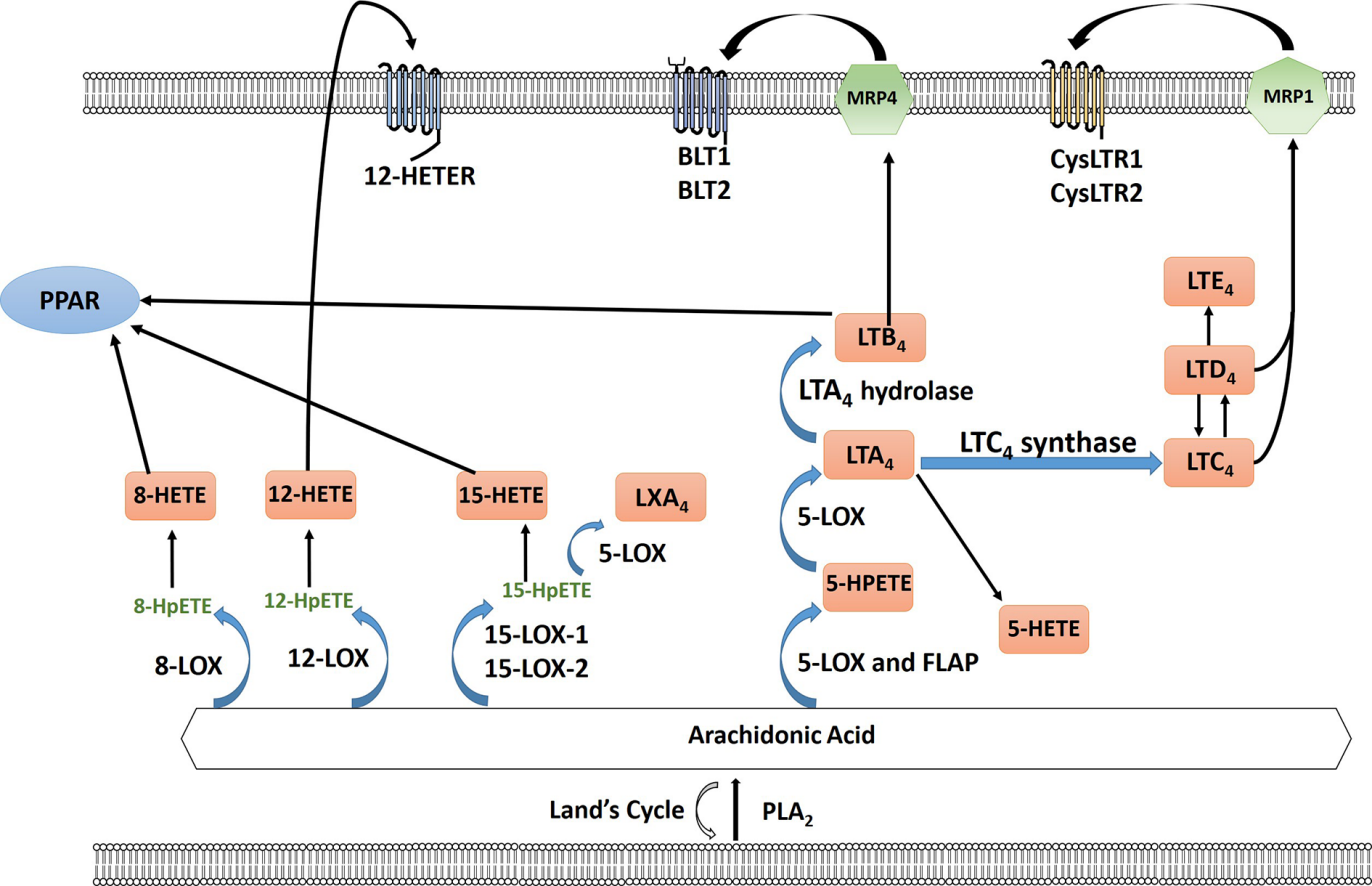
Arachidonic acid (AA) is liberated from the plasma membrane *via* phospholipase A2 (PLA_2_). AA can be recycled by the Lands cycle, which is a reacylation/deacylation cycle, that serves to keep the concentration of free AA at a low level. AA is converted by different lipoxygenase enzymes to form either hydroperoxyeicosatetraenoic acid (HPETEs) which is then reduced to the corresponding hydroxy compound. 12-HETE can signal through the G-protein-coupled receptor 12-HETE (12-HETER). 8-HETE, 15-HETE and leukotriene LTB_4_ can signal through PPAR. 5-LOX and 5-lipoxygenase activating protein (FLAP) convert AA to leukotrienes through a series of reactions using 5-LOX and as well as LTA_4_ hydrolase and LTC_4_ synthase including LTA_4_, LTB_4_, LTC_4_, LTD_4_, and LTE_4_. LTB_4_ is exported *via* multidrug resistance protein 4 (MRP4) to signal through leukotriene B4 receptor BLT1/2. LTC_4_ and LTD_4_ is exported *via* multidrug resistance protein 1 (MRP1) to bind to cysteinyl leukotriene receptor 1 or 2 (CysLTR1/2).

The cyclooxygenase (COX) pathway converts arachidonic acid to PGH_2_ which is then further converted to 5 different eicosanoids, prostaglandin E_2_ (PGE_2_), prostaglandin F_2α_ (PGF_2α_), prostaglandin D_2_ (PGD_2_), Prostaglandin I_2_ (PGI_2_) (prostacyclin), and thromboxane (TXA_2_) by specific PG synthases which are then exported to signal through their cognate receptors (Wang and DuBois, 2010; Reader et al., 2011) (**Figure 1**). The COX pathway has been the main focus of research especially concerning PGE_2_. PGE_2_ binds to four different G protein-coupled receptors EP1-4 which signal to different downstream signaling pathways (Reader et al., 2011). In the lipoxygenase pathway both HETEs and leukotrienes are produced by multiple subfamilies of LOX enzymes: 5-, 8-, 12-, 15-LOX. 5- (ALOX5) and 12-LOX (ALOX12) have been reported to have pro-carcinogenic roles, whereas 15-LOX-2 (ALOX15B) may have an anticancer effect (Guo et al., 2011). The role of 15-LOX-1 (ALOX15) is controversial (as reviewed in Guo et al., 2011). The two major isoforms of 12-LOX are platelet (p12-LOX) and leukocyte (l12-LOX) LOX with pLOX defined as the main 12-LOX in humans. 12-LOX catalyzes the stereospecific oxygenation of AA to form 12(S)-hydroperoxyeicosatetraenoic acid (HPETE), which is converted to 12(S)-hydroxyeicosatetraenoic acid (12-HETE) (Wang and DuBois, 2010; Guo et al., 2011) (**Figure 2**). It has been hypothesized that HETEs can bind to GPCRs, with 3 identified thus far, and at higher concentrations they can also activate nuclear transcription factors peroxisome proliferator-activated receptors (PPARs) (Panigrahy et al., 2010; Choi and Bothwell, 2012; Powell and Rokach, 2015; Garcia et al., 2017; Hoxha and Zappacosta, 2020). In addition to HETEs, 5-LOX, and 5-LOX activating protein (FLAP) convert AA to a series of leukotrienes including LTA_4_, LTB_4_, LTC_4_, LTD_4_, and LTE_4_ that when exported can signal through cognate receptors including leukotriene B4 receptors (BLT) and cysteinyl leukotriene receptors (CysLTR) (Wang and DuBois, 2010).

**Figure 3:**
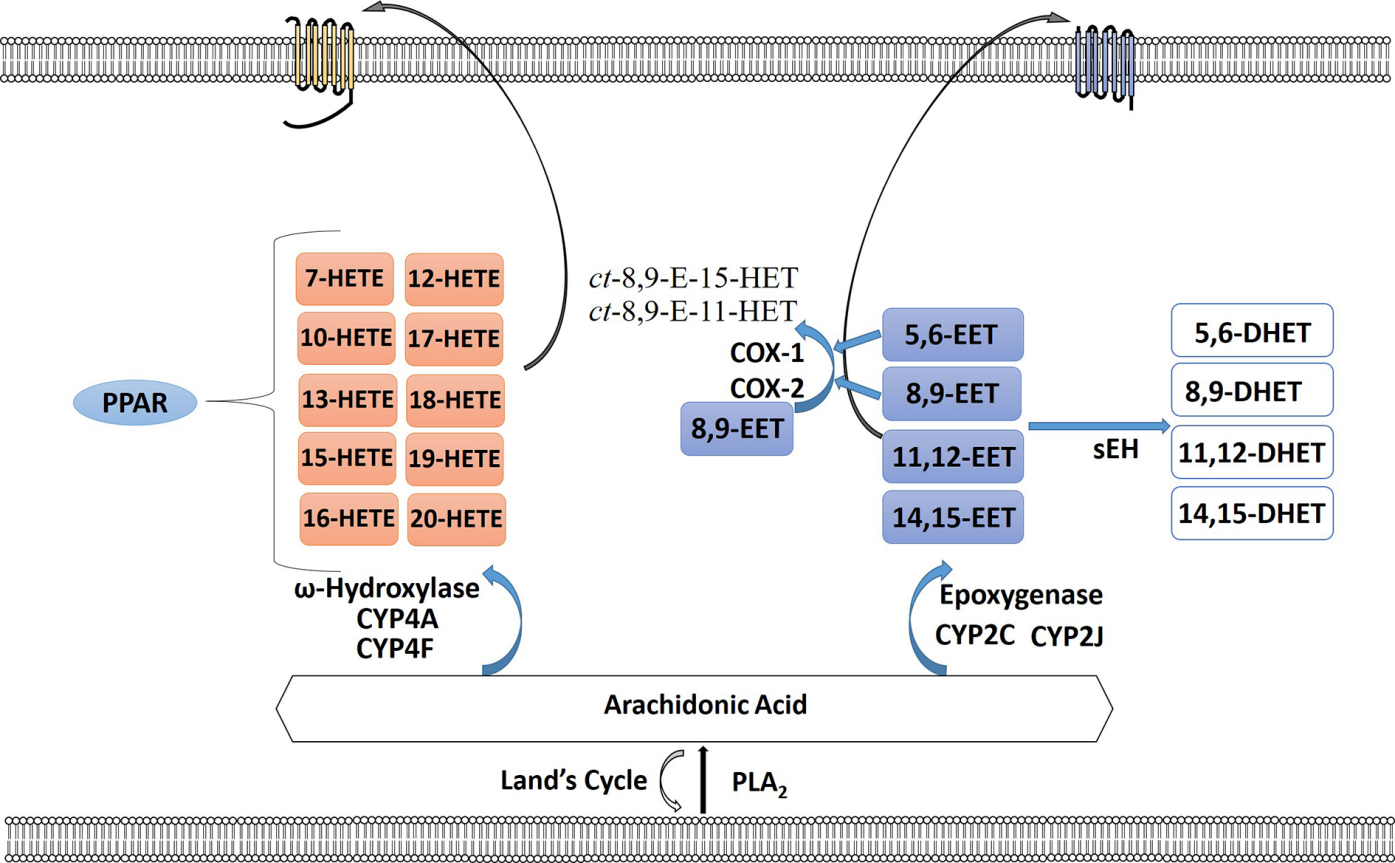
The cytochrome P450 epoxygenase pathway creates a series of regioisomeric epoxyeicosatrienoic acids (EETs) or hydroperoxyeicosatetraenoic (HETEs). Arachidonic acid (AA) is metabolized by the CYP ω-hydroxylases to 7-, 10-, 12-, 13-, 15-, 16-, 17-, 18-, 19-, and 20-HETEs. HETEs can bind to PPAR intracellularly or bind their receptor on the plasma membrane surface. EETs are metabolized by soluble epoxide hydrolase (sEH) to form the corresponding fatty acid diols. 5,6-EET and 8,9-EET are also substrates for COX-1 and COX-2 forming *ct*-8,9-E-11-HET and *ct*-8,9-E-15-HET. EETs can also signal through their cognate receptors.

The cytochrome P450 (CYP) epoxygenase pathway, a largely neglected pathway in cancer, leads to the creation of regioisomeric epoxyeicosatrienoic acids (EETs) or HETEs (Panigrahy et al., 2010; Panigrahy et al., 2011). EETs are then metabolized by soluble epoxide hydrolase (sEH) to form the corresponding fatty acid diols (Wang et al., 2019) (**Figure 3**). Arachidonic acid is metabolized by the CYP ω-hydroxylases to 7-, 10-, 12-, 13-, 15-, 16-, 17-, 18-, 19-, and 20-HETEs, the principal metabolite being the pro-inflammatory 20-HETE (Panigrahy et al., 2010). Although EETs are primarily metabolized by sEH, a few studies have observed that the 5,6-EET and 8,9-EET are but substrates for COX-1 and COX-2 (Zhang et al., 1992; Carroll et al., 1993; Moreland et al., 2007; Rand et al., 2017). Three EET regioisomers were found to be substrates for COX, and EET substrate preference for both COX-1 and COX-2 were estimated as 8,9-EET > 5,6-EET > 11,12-EET, whereas 14,15-EET was inactive. 8,9-EET is metabolized by COX to form ct-8,9-E-11-HET (8,9,11-EHET) and ct-8,9-E-15-HET (8,9,15-EHET) (Choi and Bothwell, 2012; Rand et al., 2017; Rand et al., 2019). Receptors for EETs have only recently been identified. A low affinity receptor for 11,12-EET, GPR40, has been identified in vascular cells that upon stimulation can lead to increase in Cx43 and COX-2 expression in endothelial cells *via* ERK phosphorylation (Park et al., 2018). A high affinity binding protein that may be a receptor was identified in smooth muscle, endothelial and U937 cells that can bind 8,9-EET, 11,12-EET, and 14,15-EET (Chen et al., 2011). Is it not yet known if these receptors play a role in gynecological malignancies.

## Eicosanoids in Ovarian Cancer

In the United States, ovarian cancer is the second most common gynecologic cancer and leads to more deaths than any other cancer of the female reproductive tract (Desai et al., 2014; Torre et al., 2018). Epithelial ovarian cancer encompasses a heterogenous group that is differentiated by cell, site of origin, pathological grade, risk factors, prognosis, and treatment (Torre et al., 2018). And of those, epithelial ovarian cancer accounts for 90% of all ovarian cancer cases (Torre et al., 2018). Epithelial malignancies are further subdivided into type I or type II based on clinicopathologic features and molecular features. Type I ovarian cancers, with the exception of clear cell, are considered low grade and are thought to usually develop from extraovarian benign lesions that embed within the ovary. Type II ovarian cancers are high grade characterized by aggressive behavior, late stage at diagnosis and low survival (Prahm et al., 2015; Torre et al., 2018). Clear cell, mucinous, low grade endometrioid and low grade serous cancers are type I, whereas high grade serous, high grade endometrioid, carcinosarcomas, and undifferentiated cancers are type II tumors (Jayson et al., 2014; Nezhat et al., 2015; Beeghly-Fadiel et al., 2018).

### Epoxygenase and Lipoxygenase Pathways in Ovarian Cancer

While the cyclooxygenase pathway is the most studied pathway in ovarian cancer, several studies have identified a role for the CYP P450 epoxygenase pathway. HETEs produced by CYP mono-oxygenases have been implicated in cancer development, particularly 20-HETE (Chen et al., 2005; Guo et al., 2006; Guo et al., 2007; Alexanian et al., 2009; Alexanian et al., 2012). Alexanian and colleagues have shown that CYP4A/4F genes were increased in ovarian cancer tissue compared to normal tissues and further more CYP4F2 protein and 20-HETE were also higher in ovarian cancer samples (Alexanian et al., 2012) thus demonstrating a potential role for 20-HETE in ovarian cancer which warrant additional mechanistic studies for this pathway.

The lipoxygenase pathway has been identified to play a role in ovarian cancer growth and progression (**Figure 2**). Women with increased levels of 8-HETE produced by 8-LOX and other free fatty acid metabolites were at higher risk of developing ovarian cancer in the ensuing decade implicating a role for inflammation in the initiation and promotion of ovarian cancer (Hada et al., 2019). 12-LOX expression is higher in high grade serous ovarian cancer compared to normal ovarian tissue. Ovarian cancer cell lines with increased expression of 12-LOX had increased production of 12-HETE. The addition of 12-HETE to ovarian cancer cells leads to an increase in cellular proliferation (Guo et al., 2011). 12-LOX-12-HETE pathway also inhibits apoptosis in ovarian cancer through activation of NF-kB pathway (Liu Q. et al., 2018). The 5-LOX enzyme leads to the creation of both 5-HETE and several leukotrienes. Wen and colleagues reported that hypoxia leads to an increase in 5-LOX metabolites and promotes migration and invasion of tumor associated macrophages and upregulation of MMP-7 (Wen et al., 2015). In cisplatin-resistant ovarian cancer cell line SKOV-3, upregulation of leukotriene B4 and its receptor leukotriene B4 receptor-2 (BLT2) leads to activation of STAT-3 and IL-6 and depletion of BLT2 increased cell sensitivity to cisplatin chemotherapy (Park et al., 2016). In addition, BLT2 also plays a key role in ovarian cancer invasiveness and metastasis (Seo et al., 2012). Transcriptomic analysis of tumor cells and tumor-associated macrophages (TAMs) from ascites identified a synergistic relationship between arachidonic acid, cytokines and disease recurrence (Reinartz et al., 2016). A network of lipid mediators between tumor cells and TAMs were identified which includes products of phospholipid hydrolysis, prostanoids and products of lipoxygenase pathway (Reinartz et al., 2016). These studies highlight the lipoxygenase pathway in modulation of both the tumor microenvironment as well as directly affecting the ovarian tumor cells in the progression of ovarian tumorigenesis.

### Cyclooxygenase Pathway in Ovarian Cancer

The cyclooxygenase pathway is the most investigated pathway in ovarian cancers with studies identifying both COX-1 and COX-2 as potential mediators of pro-tumorigenic prostaglandin production. There has been conflicting evidence as to whether COX-1 or COX-2 plays a larger role in ovarian cancer tumorigenesis. Denkert et al. (2002) found that COX-2 expression was an independent prognostic factor for poor survival in ovarian surface epithelial tumors. Various groups have reported differences in COX-1 vs COX-2 expression in different ovarian cancer histological subtypes. Epithelial ovarian cancer demonstrates a high rate of COX-1 and COX-2 expression especially in non-mucinous tumors and a combination of high COX-1 but low COX-2 was associated with poor progression-free and overall survival (Khunnarong et al., 2010). COX-1 was found to be overexpressed in high grade serous ovarian tumors; whereas, COX-2 was highly expressed in endometrioid and mucinous tumors (Wilson et al., 2015). In a follow up study, COX-1 expression was higher in low and high grade serous tumors and type II tumors in comparison to type I tumors while COX-2 was more highly expressed in non-serous and type I tumors (Beeghly-Fadiel et al., 2018).

The association of elevated prostaglandins in the tissues and ascites from ovarian cancer was first reported almost 40 years ago. Prostaglandins PGE_2_, PGF_2α_ and 6-keto-PGF_1α_ (a degradation product of PGI_2_) were measured in advanced human ovarian cancer tissues (Bauknecht et al., 1985). In tumors that were resistant to chemotherapy, higher levels of these prostaglandins were detected compared to chemotherapy responsive tumors (Bauknecht et al., 1985). Ascitic fluids from ovarian cancer patients were compared to patients with benign gynecologic conditions and higher amounts of fatty acid palmitoleic acid were measured in cancer patients (Punnonen et al., 1986). Concentrations of PGE_2_, 6-keto-PGF_1α_, TxB_2_, cyclic adenosine monophosphate (cAMP) and cyclic guanosine monophosphate (cGMP) in ovarian tumor tissue were measured in 38 post-menopausal women with malignant or benign ovarian tumors and in six women without ovarian neoplasms (Heinonen and Metsä-Ketelä, 1988). PGE_2_ and TXB_2_ contents in ovarian cancer tissue were significantly (P < 0.05) higher than in normal ovarian tissue (Heinonen and Metsä-Ketelä, 1988). Ovarian cancer is more common in patients with elevated gonadotropins such as FSH and LH and they are detectable in ovarian tumor fluid. FSH/LH lead to increased PGE_2_ production through upregulation of the expression of COX-1 and COX-2 and activation of the PI3K/AKT pathway that ultimately leads to tumor cell migration and invasion (Lau et al., 2010).

#### Phospholipase A_2_ in Ovarian Cancer

Phospholipase A_2_ enzymes, which convert lipids to arachidonic acid, the substrate for the COX enzyme, have been implicated in several cancers. EOC ascites contains high levels of oncogenic lipid growth factors including lipid products produced by PLA_2_ such as lysophosphatidic acid (LPA) and lysophosphatidylcholine (LPC) (Cai et al., 2012). Human EOC ascites contained microvesicle-free cPLA_2_ and iPLA_2_ that were secreted in an ABC transporter dependent mechanism. Chen et al. (2018) reported that the ABCC1 inhibitor MK571 was the most effective inhibitor of PLA_2_ activity. However, ABCC4 is also inhibited by MK571 and we have found increased expression of ABCC4 (MRP4), the exporter for PGE_2_, to be overexpressed in multiple ovarian cancer cell lines and tumor tissue; therefore, it is possible that ABCC4 could also play a role in PLA_2_ secretion (Kochel and Fulton, 2015; Kochel et al., 2017; Ching et al., 2018). Treatment of ovarian cancer cells with chemotherapy drug VP16 causes release of AA resulting in elevation of PGE_2_ levels and enhancing the repopulation of ovarian cancer cells (Zhao et al., 2017). Berberine can block the caspase 3‐iPLA_2_‐AA‐COX‐2‐PGE_2_ pathway by inhibiting the expression of iPLA_2_ and COX‐2. PGE_2_ can lead to increased phosphorylation of FAK. Treatment with berberine reverse PGE_2_ induced FAK phosphorylation thereby leading to inhibition of VP16 induced ovarian cancer cell repopulation (Cui et al., 2017; Zhao et al., 2017). Targeting of cytosolic phospholipase A_2_ can increase efficacy of low-dose radiotherapy in ovarian cancer (Schulte et al., 2011). These studies demonstrate the role of PLA_2_ in treatment response and tumor promoting activities such as proliferation, migration and invasion and tumor growth *in vivo* (Schulte et al., 2011; Cai et al., 2012; Chen et al., 2018).

#### Prostanoids in Ovarian Cancer

PGE_2_ signals through four receptors of which two have been identified to play a role in ovarian cancer (Spinella et al., 2004a; Ching et al., 2018; Zhang et al., 2019). There is increased expression of EP4 in ovarian cancer cell lines and primary tumor tissue compared to normal ovarian cell lines and tissue (Ching et al., 2018; Fan et al., 2018). Treatment of SKOV-3 and MDAH-2774 with dmPGE_2_ lead to a dose dependent increase in COX-2, Bcl-2 and bax expression as well as an increase in proliferation and decrease in apoptosis (Munkarah et al., 2002). Bagnato’s group studied the relationship between endothelin-1, COX and EP receptors in ovarian cancer growth and invasion (Spinella et al., 2004b; Spinella et al., 2004a; Spinella et al., 2004c). Activation of the endothelian A receptor (ET_A_R) by endothelin-1 (ET-1) leads to an increase in COX-1 and COX-2 expression as well as PGE_2_ production (Spinella et al., 2004b). ET-1 induced PGE_2_ production then signals through EP2 and EP4 to stimulate VEGF production. Additionally, signaling through the EP4 receptor leads to an increase in ovarian cancer cell invasiveness (Spinella et al., 2004a). Signaling through the EP2 receptor leads to an increase in proliferation and invasion through the NFkB pathway (Zhang et al., 2019).

There is conflicting evidence on whether PGI_2_ is anti- or pro-tumoral prostanoid. PGI_2_ analog iloprost has been shown to have anti-inflammatory and anti-tumoral effects in lung cancer (Lo et al., 1998; Tennis et al., 2010). In ovarian cancer, iloprost inhibited migration and invasion through downregulation of MMP-2 expression (Ahn et al., 2018). In study using engineered OSE cells to form ovarian tumors, tumors demonstrated an increase in COX-1 expression but not COX-2 (Daikoku et al., 2005). Additionally, PGI1, not PGE2, was the major prostaglandin produced by these tumors (Daikoku et al., 2005). Treatment with COX-1 inhibitor SC-560 reduced cell proliferation and accelerated apoptosis; whereas, celecoxib had little effect on tumor growth. SC-560 also decreased PGI_2_ production by OSE cells and tumors. In a follow up study, Daikoku et al. (2007) demonstrated that aspirin (Cox-1 inhibitor) not only lowered PGI_2_ levels in T2 and OVCAR-3 cells, but it also attenuates the transactivation of PPARδ, which indicates the inhibition of PG production and PPARδ are downstream targets of COX-1 PGs. This suppression of COX-1- PPARδ signaling by aspirin lead to compromised tumor growth in mice; this result suggests the importance of this pathway to stimulate tumor growth and offers a promising target for ovarian cancer treatment (Daikoku et al., 2007).

Lipid mediators play an important role in both platelet and ovarian cancer functions. Tumor cells can act as agonists for platelets leading to an increase in platelet adhesion, aggregation and degranulation (Serhan et al., 2018). Meanwhile platelets can protect tumor cells from host defenses and increase tumor cell invasion and extravasation (Serhan et al., 2018). Lipid mediators that act as platelet agonists are often produced by tumor cells and are found in the tumor microenvironment. TXA_2_, PGD_2_, PGE_2_, and PGI_2_ are all important prostanoids for platelet function including activation of platelets, TXA_2_ and PGE_2_, or inhibiting platelet aggregation, PGI_2_ or PGD_2_ (Friedman et al., 2015; Contursi et al., 2017; Serhan et al., 2018). PGE_2_ and prostacyclin are found in the ovarian tumor microenvironment linking lipid signaling and platelets in ovarian cancer (Reinartz et al., 2016). In ovarian cancer patients with residual disease, plasma 6-keto-PGF1α (a metabolite of PGI_2_) in cancer patients (146.7 +/− 14.7 pg/ml, mean +/− SE) was higher (P less than 0.02) than that in the controls (85.3 +/− 9.2 pg/ml, n = 17). Also, the release of TXB_2_ (a metabolite of TXA_2_) during spontaneous clotting of the blood samples was greater in the patients than controls (Ylikorkala et al., 1983).

Cyclopentenone prostaglandins of the A and J series, PGA_2_, PGA_1_, and PGJ_2_, which are produced by dehydration of PGE_2_, PGE_1_, and PGD_2_, respectively, have various biological activities including both pro- (Oliva et al., 2003) and anti-tumoral effects (Sasaki and Fukushima, 1994; Straus and Glass, 2001). Sasaki demonstrated that ovarian cancer cell lines resistant to cisplatin, doxorubicin and L-phenylalanine mustard were sensitive to antitumor prostaglandins delta-7-prostaglandin A1 (d7-PGA_1_) and delta-12-prostaglandin J2 (d-12-PGJ_2_) (Sasaki et al., 1991) and a derivative of d7-PGA_1_, 13,14-dihydro-15-deoxy-delta7-prostaglandin-A1-methyl ester, exhibits antitumor activities against cisplatin resistant ovarian cancer cells (Sasaki et al., 1999). McClay and colleagues showed that d-12-PGJ_2_ can synergize with cisplatin and radiation thus increasing the efficacy of these treatments against ovarian cancer (McClay et al., 1996). 15-deoxy-delta12,14-prostaglandin J2 (15-d-PGJ_2_) has been shown to strongly induce apoptosis and autophagic cell death pathways and inhibit sirtuin in chemotherapy resistant cell lines (Jong et al., 2011; Tae et al., 2018). Interestingly, expression of PGD_2_ in high-grade serous ovarian cancer is an independent marker of good prognosis and is associated with increase in disease-free survival, the absence of relapse and sensitivity to platinum-based therapy (Alves et al., 2019) which further links the antitumor properties associated with the PGJ prostaglandins which are derived from PGD_2_. Given the activity of these metabolites against drug resistant ovarian cancer cell lines, which is what ultimately leads to mortality in patients, warrants additional studies on these compounds.

#### COX Enzymes in Ovarian Cancer

Several studies have been published supporting the role of COX-1 in ovarian cancer development and progression. Gupta et al. (2003) found increased expression of COX-1 mRNA and protein in ovarian cancer tissues compared to normal ovarian tissue. They also examined the link between the COX pathway and angiogenesis; ovarian epithelial cells with high COX-1 expression exhibit high levels of transcription factors including HIF-1α, VEGF, VEGF receptor: Flk-1. In a follow up study, a genetically engineered mouse model of ovarian EOC was used to evaluate whether COX-1 expression is linked to mutations in tumor suppressors *p53* and *Rb* (Daikoku et al., 2006). In mice with these mutations, high COX-1 mRNA levels were detected in well differentiated serous epithelial neoplasms; COX-2 levels remain to be undetectable and this is consistent *in situ* tumor samples (Daikoku et al., 2006). In a follow up study, peroxisome proliferator-activated receptor δ (PPARδ), a downstream target of COX-1, is directly activated by PGI_2_ and transactivated by PGE_2_, stimulates cell proliferation and tumor growth in cancers like mammary and hepatocellular, and was found to be overexpressed in mouse ovarian cancer cell lines (Daikoku et al., 2007). COX-1 has been identified to also produce prostaglandins in ovarian cancer cell lines. Increased levels of COX-1 were identified in 3 out of 10 ovarian cancer cell lines, PGE_2_ production was also elevated in those cell lines (Kino et al., 2005). Non-selective COX inhibitor (indomethacin) and a selective COX-1 inhibitor (SC-569) significantly decreased PGE_2_ production; however, COX-2 inhibitors, NS-398 and rofecoxib, did not affect PGE_2_ production which suggests that COX-1 is regulating the production of PGE_2_ in these cell lines (Gupta et al., 2003; Kino et al., 2005; Rask et al., 2006).

In addition to COX-1, COX-2 has also been shown to play a role in ovarian cancer progression. COX-2 is constitutively expressed in various EOC cell lines; introduction of PGE_2_ increases COX-2 expression, increases proliferation as well as reduces apoptosis (Munkarah et al., 2002). COX-2 derived PGE_2_ also promotes ovarian cancer cell invasion through an epidermal growth factor (EGF) signaling mechanism in which a positive feedback loop leads to increases in COX-2, PGE_2_ and EGF (Qiu et al., 2014). COX-2 can also lead to an increase in pro-angiogenic proteins. The expression of nerve growth factor (NGF) is frequently overexpressed in EOC leading to an increase in VEGF, β-catenin/TCF-Lef, survivin, and MYC. Garrido et al. (2019) identified that this was due to a NGF-regulated increase in COX-2/PGE_2_ signaling. Dursun et al. (2005) hypothesize that the difference between invasive ovarian tumors and borderline ovarian tumors, which are less threatening in terms of survival, is the level of COX-2 expression. Overexpression of COX-2 stimulates prostaglandin production and cellular growth, reduces apoptosis enzymes, promotes mutagenesis, tumorigenesis, and angiogenesis (Dursun et al., 2005). COX-2 positive ovarian cancer cases also have higher expression of MDR1/P-glycoprotein (P-gp). P-gp is a drug efflux pump whose presence is associated with unfavorable significance in ovarian cancer treated with chemotherapy and may be upregulated by COX-2. Previously, Denkert et al. (2002) demonstrated that expression of COX-2 is an independent unfavorable prognostic factor in ovarian cancers. In a follow up study, they have extended these findings to demonstrate a positive correlation between COX-2 and P-gp expression in human ovarian carcinomas and unfavorable prognostic and predictive significance in tumors expressing COX-2 and/or P-gp (Surowiak et al., 2006). Combination treatment with cisplatin or docetaxel with NSAID, NS-398 or sulindac, can lead to dose-dependent enhancement of cytotoxicity which could be enhanced under hyperthermic conditions (41°C) (Barnes et al., 2007). Denkert et al. (2003) investigated COX enzyme dependent vs independent effects of the NSAID NS-398 on ovarian cancer cell lines that are either positive for COX-1/2, OVCAR-3, or negative for COX-1/2 expression, SKOV-3. They demonstrated that NS-398 could inhibit proliferation through induction of cell cycle arrest that is independent of COX-2 inhibition and the inhibitory effect could not be rescued with the addition of PGE_2_ (Denkert et al., 2003).

COX inhibitors have been explored as both a monotherapy and as well in combination with chemotherapeutics. Li and colleagues investigated the effect of COX-1 and COX-2 inhibitors as a monotherapy and as well as in combination with taxol on SKOV-3 xenograft mouse model (Li et al., 2008; Li et al., 2010; Li et al., 2011; Li et al., 2012a; Li et al., 2012b; Li et al., 2013). Nimesulide (COX-2 inhibitor) treatment of mice with SKOV-3 xenograft tumors leads to a reduction in microvessel density (MVD) and PGE_2_ levels but did not significantly decrease tumor growth (Li et al., 2008). This led Li to examine the effects of COX-1 inhibitor, SC-560, and COX-2 inhibitor, celecoxib, or dual inhibitor, indomethacin, on xenograft tumor growth. Monotherapy treatment was not effective in decreasing tumor volume; however, dual SC-560 and celecoxib inhibitor significantly reduced relative tumor volume (Li et al., 2012a). Single treatment with SC-560 or celecoxib also significantly prolonged survival of the mice compared to control. Previously, Gupta et al. (2003) reported that COX-1 and pro-angiogenic proteins were overexpressed in ovarian tumors and treatment with COX-1 inhibitor SC-560 but not COX-2 inhibitor celecoxib lead to a decrease in secretion of VEGF by OVCAR-3 cells *in vitro*. Li found similar results in their *in vivo* studies (Li et al., 2012a). Li and colleagues also investigated the combination of SC-560 and taxol as well as celecoxib and taxol on the effect of SKOV-3 tumor MVD, apoptosis and VEGF transcript levels (Li et al., 2012a). Taxol in combination with SC-560 was superior to taxol with celecoxib and while the authors did not provide an explanation for the observed differences in the two inhibitors, the potential remains for the possibility of off target or non-COX related effects of the inhibitors (Li et al., 2012a; Li et al., 2013). Using a combination of siRNAs against COX-1/COX-2, Denkert et al. (2003) examined the role COX-2-dependent and COX-2 independent effects of COX inhibitors on ovarian cancer proliferation. As opposed to the predominant role of COX-1 in SKOV-3 cells, in OVCAR-3 cells, COX-2 was responsible for PGE_2_ production and production could be inhibited with COX-2 inhibitor NS-398. COX-1/COX-2 inhibitors lead to a decrease in proliferation that was independent of COX activity and expression through induction of Go/G1 cell cycle arrest. Uddin et al. (2010) examined the molecular pathways involved in COX-2 inhibition in ovarian cancer. COX-2, but not COX-1, expression was associated with expression of pAKT in ovarian cancer tumor tissue. In MDAH2774 and SKOV3 cell lines, aspirin and NS-398 also lead to a decrease in proliferation and induction of apoptosis. While the authors did not explore specific EP receptors in this study, EP4 has been shown to activate PI3K/AKT (Hsu et al., 2017). Given the results of these studies the possibility exists that COX independent effects play a significant role in the function of COX inhibitors.

#### Cyclooxygenase in Ovarian Cancer Tumor Microenvironment

The innate and adaptive immune system plays an essential role in the progression and spread of cancer. The ovarian cancer microenvironment is highly immune suppressive (Preston et al., 2011). Natural killer cells (NK), part of the innate immune system, can lyse cells without first having to recognize specific antigens and have been reported to play an essential role in controlling metastasis in breast cancer with contradictory roles in ovarian cancer (Dong et al., 2006; Holt et al., 2012; Sun et al., 2018). Myeloid-derived suppressor cells (MDSC), which are a heterogenous population of macrophages, DCs and granulocytes at early stages of differentiation, is another driver of immune suppression with the ability to block local and systemic immune activation (Preston et al., 2011). TAMs are one of the most abundant immune cells in the tumor microenvironment (Colvin, 2014). These heterogenous cells can act to either stimulate or suppress the immune response and these opposing behaviors have been classified as either classically activated, M1, or alternatively activated, M2 (Colvin, 2014). M1-polarized macrophages produce pro-inflammatory and immunostimulatory cytokines, are involved in T_H_1 responses and are considered cancer killing; whereas, M2-polarized macrophages are immunosuppressive, associated with the T_H_2 response and are considered cancer promoting (Colvin, 2014). Dendritic cells (DC) can either activate adaptive immunity or tolerize T cells specific for tumor-associated antigens (Pardoll, 2015). Cytotoxic T cells (CTL) exhibit antitumor immune functions with CD8+ cytotoxic T cells generally are the primary mediators of antitumor immune response. Helper T cells, which are required to activate cytotoxic T cells, are functionally divided into subsets including T_H_1 and T_H_2 according to secretory cytokines and immunologic roles (Lee et al., 2019). The T_H_1/T_H_2 balance plays an essential role in immune response to cancer with T_H_1 eliciting an anti-tumor response whereas T_H_2 is pro-tumorigenic (Burkholder et al., 2014).

Mucin molecules (MUCs) are aberrantly secreted from ovarian cancer cells. There is a positive correlation with five year survival rate in patients with high density of M1 TAMS and a high M1/M2 ratio and therapeutic targeting against M2 inhibits disease progression (Luo et al., 2006; Mantovani et al., 2006; He et al., 2013). He et al. showed that an increased concentration of COX-2+ cancer cells, decreased ratio of M1/M2 TAMs, and an increased concentration of COX-2+ TAMs was associated with poor prognosis (He et al., 2013). Populations of M2 TAMs and COX-2+ TAMs frequently converged. There was also an association between COX-2+ cancer cells and COX-2+ TAMs (He et al., 2013). Expression of MUC2 can result in M2-polarization and induce TAM COX-2 expression. The polarization of M2 TAMs and expression of COX-2 can lead to an increase in PGE_2_ in the microenvironment by TAMs and cancer cells which can lead to an acceleration of ovarian cancer progression (He et al., 2013).

MDSCs expand during cancer and can suppress T-cell facilitated responses (Gabrilovich and Nagaraj, 2009; Ostrand-Rosenberg and Sinha, 2009; Rodríguez-Ubreva et al., 2017). Myeloid cell differentiation is highly dependent on DNA methylation changes and pro-inflammatory mediators such as PGE_2_ regulate MDSC accumulation (Sinha et al., 2007; Álvarez-Errico et al., 2015; Rodríguez-Ubreva et al., 2017). Rodríguez-Ubreva et al. (2017) demonstrated that ovarian cancer cells can induce differentiation of monocytes to MDSCs by causing hypermethylation of MDSC specific genes by signaling through the PGE_2_ EP2 and EP4 receptors leading to upregulation of DNMT3A. DCs and MDSCs show opposing roles in the immune system, suppression of DCs contributes to cancer progression while MDSCs suppress the ability of CD8+ T cells to mediate effective anti-tumor response (Rodríguez-Ubreva et al., 2017). COX-2-PGE_2_ leads to positive feedback signaling that results in blocking CD1α+ DC differentiation and leading to induction of CD14+CD33+CD34+ monocytic MDSCs (Obermajer et al., 2011). The frequency of CD11b(+)CD33(+) MDSCs in ovarian cancer are closely correlated with local PGE_2_ production. Inhibition of EP2 and EP4 or interruption of COX-2-PGE_2_ feedback using COX-2 inhibitors decreases the production of suppressive mediators and inhibitory functions of MDSCs from cancer patients.

Wong et al. (2016) observed an unexpected increase in both type-1 mediators, IFNγ and TNFα, induction of immune suppressive COX-2 and hyperactivation of MDSCs in the tumor microenvironment of ovarian cancer patients. The synergistic action of both IFNγ and TNFα which lead to increased MDSC activity and expression of IDO1, NOX2, IL-10 and COX-2 mRNA and protein was dependent on COX-2-PGE_2_ such that blockade of COX-2 was able to completely reverse enhanced suppressive ability of the hyperactivated MDSCs (Wong et al., 2016). Blockade of COX-2 or the EP4 receptor reduces expression of IDO1, an immunosuppressive enzyme, leading to an increase in CD3+ and CD8+ cells thus increasing T cells and control of tumor growth (Hennequart et al., 2017). While IL-10 and PGE_2_ were identified as having immunosuppressive capabilities in ascites from ovarian cancer patients, PGE_2_ was found to be the more important of the two that leads to impaired T cell stimulatory capacity of DC cells (Brencicova et al., 2017). Cytotoxic T cells directed against endogenously expressed antigens are key for antigen-specific cancer immunotherapy. COX-2-PGE_2_ was identified as resistance factors for suppression of antigen-induced interferon-gamma secretion of T cells and generation of factors that suppress the immune system leading to escape from immune surveillance and resistance to cellular immunotherapy (Göbel et al., 2014).

Maintenance of CTL, T_H_1 and NK cell-mediated type I immunity is key for effective antitumor responses. Liu et al. (2009) analyzed the pattern of CD8+ (CTL), CD57+ (NK), and CD1α+ (DC) infiltrating immune cells in intraepithelial or stromal spaces with survival and COX-1 and COX-2 expression. PGE_2_ can alter the T_H_1/T_H_2 balance, suppress DC function, and increase the number of tumor-infiltrating regulatory T cells (Liu et al., 2009). Higher levels of COX-2 expression occurred more frequently in serous ovarian carcinoma compared to other types (P<0.05) and higher levels of COX-1 or COX-2 tended to correlate with poor prognosis (n.s.) (Liu et al., 2009). COX may impact ovarian cancer prognosis and the pattern of tumor-infiltrating immune cells. Ovarian cancers were analyzed by hierarchical cluster analysis based on the number of tumor-infiltering immune cells and three different clusters were generated (Liu et al., 2009). Cluster 1 was characterized with the worst progression free survival and overall survival. Cluster 1 was characterized by higher expression of COX-1 and COX-2 compared to cluster 2 (P<0.05, respectively). The presence of intraepithelial CD8+ cells was negatively correlated with COX-1 and COX-2 expression (P<0.05 for both) (Liu et al., 2009). It is clear that PGE_2_ *via* COX-1/COX-2 expression plays a critical role in the ovarian cancer tumor microenvironment leading to immune suppression through multiple mechanisms. The COX/PGE2 pathway warrants additional studies including combining PGE_2_ EP4 inhibitors with immunotherapy in ovarian cancer.

## Eicosanoids in Uterine Cancer

Uterine cancer is the fourth most common malignancy as well as the most common gynecologic malignancy (Siegel et al., 2019). There are two subgroups, Type I, endometrioid cancer, which is the most common, is associated with expression of hormonal receptors and, Type 2, unrelated to estrogen, and includes histological types such as serous, clear cell, mucinous and uterine sarcomas (Creutzberg and Fleming, 2016; Di Tucci et al., 2019).

### Lipoxygenase in Uterine Cancer

The majority of the research published to date discusses the role of prostanoids in uterine cancer; however, the lipoxygenase pathway has been shown to be involved in type 2 endometrial cancer. ALOX5 (5-LOX) is generally reported to be absent in normal epithelial but is induced by pro-inflammatory stimuli and can be overexpressed in various epithelial cancers (Pidgeon et al., 2007; Wang and DuBois, 2010). In a study performed by Cummings et al. (2019), there is a significant increase in ALOX5 (5-LOX) mRNA expression in type II endometrial cancer compared to normal endometrium and elevated ALOX5 expression is associated with adverse outcomes. Given the limited number of studies available on the role of lipoxygenase as well as the lack of studies in the cytochrome P450 pathway in uterine cancer, these pathways are ones that should be evaluated in uterine cancer in the future.

### Cyclooxygenase in Uterine Cancer

Uterine leiomyosarcoma (LMS) is the second most common subtype of uterine sarcoma with uterine leiomyomas comprising the most common benign pelvic tumors in women (Sabry and Al-Hendy, 2012; Kobayashi et al., 2013). Leiomyomas release prostaglandins including 6-keto-PGF_1α_, PGF_2α_ and PGE_2_ (Rees and Turnbull, 1985). Endothelin (ET)-1 exerts survival effects in uterine leiomyoma cells and leads to increased protein expression of COX-2 and PGE_2_ production (Oyeniran and Tanfin, 2011). COX-2 expression was higher in uterine fibroids compared to healthy smooth muscle cells and selective COX-2 inhibitor celecoxib decreased fibroid cell proliferation and PGE_2_ secretion (Ke et al., 2013). Recently, in a study published by Reader et al. (2019), the role of EP4 was evaluated in LMS proliferation and migration. While minimal effects in proliferation was observed with monotherapy treatment of LMS with an EP4 inhibitor when combined with docetaxel treatment, we saw a sensitization of LMS cells to chemotherapeutic treatment (Reader et al., 2019). In addition to sensitization to chemotherapy, we also demonstrated a significant decrease in LMS cell migration. PI3K has been shown to play a significant role in the development of uterine cancer and activation of EP4 can lead to activation of the PI3K pathway (**Figure 1**) (Naumann, 2011).

One common inflammatory disease that affects approximately 10% of reproductive aged women is endometriosis, which causes chronic pain and infertility, and is defined as the presence of endometrium-like tissue outside the uterus (As-Sanie et al., 2019). Epidemiological, biological, and molecular data all indirectly suggest links between the endometriosis and endometrial cancer (Painter et al., 2018; As-Sanie et al., 2019). In 1992, Koike et al. (1992) identified increased production of 6-keto-PGF_1α_, TXB_2_, and PGE_2_ in endometrial cysts compared to non-endometrial cysts and normal ovaries. Additionally, the COX-2 pathway promotes survival, migration, and invasion of endometriotric cells and promotes angiogenesis during endometriosis progression (Banu et al., 2008; Jana et al., 2016). Inhibition of EP2 and EP4 leads to apoptosis of endometriotic cells as well as a decrease of human endometriotic epithelial and stromal cells through integrin-mediated mechanisms (Banu et al., 2009; Lee et al., 2013). As indicated in these studies a relationship exists between COX eicosanoids in the pathogenesis of endometriosis and therefore endometrial cancer.

COX-1 and COX-2 are important in normal reproduction as reviewed in (Sales and Jabbour, 2003). COX enzymes convert arachidonic acid to PGH_2_ which is then metabolized by specific synthases to thromboxanes and prostaglandins including PGE_2_ and PGF_2α_ (Wang and DuBois, 2010). PGE_2_, EP2 and EP4 receptors work in an autocrine/paracrine role in the epithelial/endothelial cell function in normal human endometrium (Milne et al., 2001). During the menstrual cycle, signaling of EP2/EP4 receptors produced a greater response in proliferative tissue compared with early and midsecretory stage tissue. COX-2 expression is up-regulated in neoplastic epithelial and endothelial cells of endometrial carcinomas (Tong et al., 2000; Jabbour et al., 2001; Ferrandina et al., 2002; Uotila et al., 2002; Jabbour et al., 2006). Lower risk of endometrial cancer was reported when aspirin and other nonsteroidal anti-inflammatory agents were used in patients which could block production of prostaglandins (Black et al., 2014). The importance of COX-2 in the early stages of endometrial cancer development was shown using a conditional endometrial-specific phosphatase and tensin homologue gene (PTEN) knockout mouse model (Daikoku et al., 2008). Dual inhibition of COX-2 and mTORC1 signaling markedly reduces endometrial cancer progression (Daikoku et al., 2014). Paclitaxel resistance may be associated with COX-2 and multidrug resistance 1 (MDR1) expression, an efflux pump that can transport a wide range of compounds including chemotherapy out of the cell, in endometrial cancer (Hasegawa et al., 2013). Co-administration of COX-2 inhibitor etodolac with paclitaxel leads to a decrease in MDR1 expression which may enhance accumulation of MDR1 substrates such as paclitaxel thus leading to an increase in paclitaxel sensitivity (Hasegawa et al., 2013). However, recently Cummings, et al. using laser capture microdissection, whole genome expression analysis and liquid chromatography-tandem mass spectrometry, did not show an increase in COX isoform expression nor PGE_2_/PGF_2α_ levels in neoplastic endometrium compared to sample matched epithelial tissue (Cummings et al., 2019). The authors do suggest that an increase in local levels of PGE_2_ and PGF_2α_ could occur due to decreased catabolism, *via* downregulation of HPGD (**Figure 1**). There was an increase in EP3 (PTGER3) and EP4 (PTGER4) receptor gene expression in type I and type II endometrial cancers which could lead to an increase in local PGE_2_-EP3/EP4 mediated signaling. In addition, our group has observed an increase in EP4 expression in primary endometriod cancer tissue (Reader et al., 2016). The differences could be due to the fact that the tissues used for this study were microdissected instead using the entire tissue for analysis as well as the use of fewer clinical samples in previous studies.

### Prostanoids in Uterine Cancer

**Figure 4:**
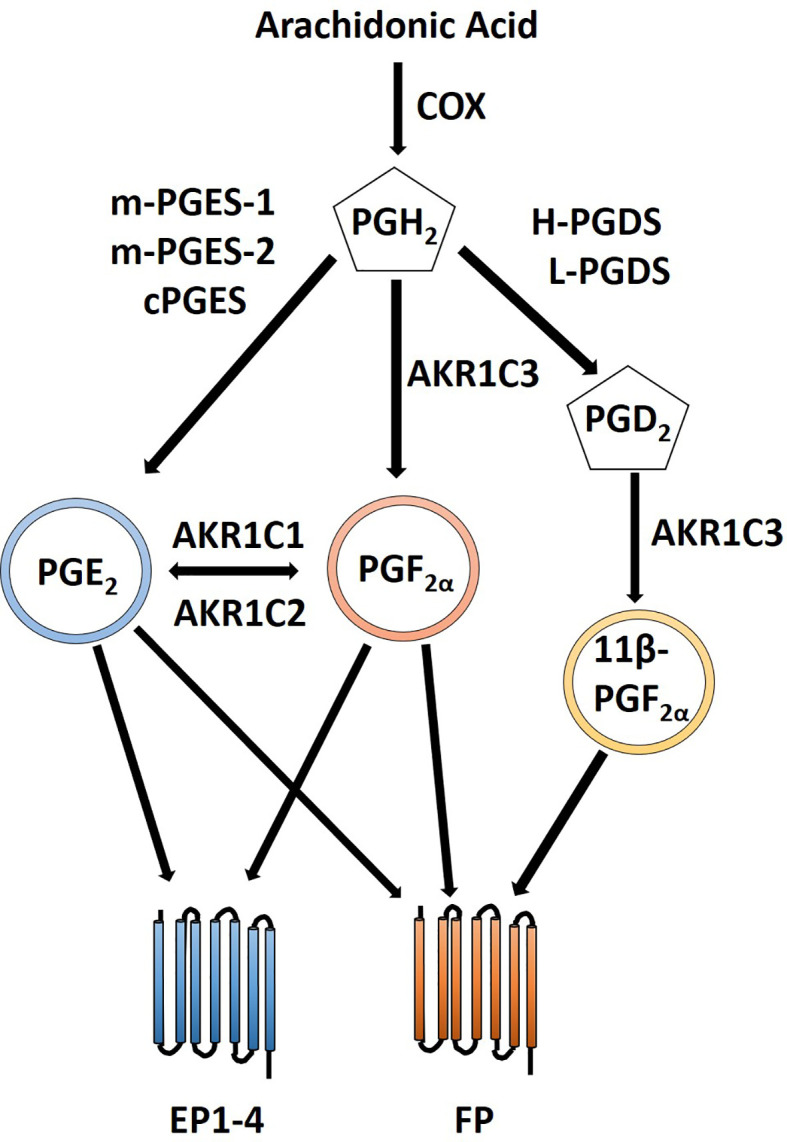
Crosstalk between PGE_2_ and PGF_2α_ prostanoid pathways. PGE_2_ is created *via* conversion of PGH_2_ by PGE synthases including m-PGES-1, m-PGES-2 and cPGES. Likewise, PGF_2α_ is formed by conversion of PGH_2_ by AKR1C3. Additionally, PGF_2α_ can be created from PGE_2_ by AKR1C1 or AKR1C2. 11β-PGF2α, a stereoisomer of PGF_2α_, can also signal *via* the FP receptor and is created by the PGD_2_ prostanoid pathway with an additional conversion by AKR1C3. PGE_2_ can signal through its cognate PGE_2_ EP receptors as well as the through PGF_2α_ FP receptor. Similarly, PGF_2α_ can signal through its FP receptor as well as through PGE_2_ EP receptors.

The F and E prostanoid pathways have been shown to contribute to endometrial cancer phenotypes including proliferation, adhesion, migration, invasion, angiogenesis, and inflammatory microenvironment by binding with their respective G-protein coupled receptors (Sales et al., 2004b; Sales et al., 2005; Sales et al., 2008a; Keightley et al., 2010b; Wallace et al., 2010; Zhu et al., 2017). PGF_2α_ exerts its action *via* FP receptors of which two isoforms exists generated *via* alternative mRNA splicing designated FPA and FPB. These isoforms are coupled to G_αq_ and can produce IP_3_ through activation of phospholipase C, intracellular calcium flux and activation of protein kinase C (Sales and Jabbour, 2003). PGF_2α_ plays an essential role in parturition and regulation of expression of the oxytocin receptor. PGE_2_ can be converted to PGF_2α_ adding a layer of crosstalk and complexity (Dozier et al., 2008) (**Figure 4**). Downstream signaling of FP and EP receptors leads to an increase in COX-2 expression (Fujino and Regan, 2003; Sales et al., 2008b). PGE_2_ can act as an agonist for FP receptors thus PGE_2_ and PGF_2α_ when locally produced by endometrial adenocarcinomas can regulate tumor cell function in an autocrine/paracrine manner by the FP receptor leading to an induction of COX-2 expression (Sales et al., 2008b). PGF_2α_ and FP receptor interaction enhances proliferation of endometrial epithelial cells (Sales et al., 2005; Sales et al., 2007). There is co-localization of COX-2 and the FP receptor within neoplastic epithelial cells of endometrial adenocarcinoma and activation of PGF_2α_-FP receptor lead to an increase in COX-2 expression through activation of ERK1/2 pathway which leads to a concomitant increase in PGF_2α_ creating a positive feedback loop (Jabbour et al., 2005). Another positive feedback involving FGF2-FGFR1 interaction that results in elevated FGF2 and COX-2 expression and enhanced proliferation *via* the FGFR1 and ERK pathways (Sales et al., 2007). In addition to proliferative changes, activation of the FP receptor can lead to an increase in adhesion, migration and invasion of endometrial adenocarcinoma cells. Alterations in cell adhesion and increased migration occur through a αVβ3-ECM dependent mechanism after PGF_2α_-FP activation (Sales et al., 2008a) and regulation of endometrial adenocarcinoma cell invasion occurs *via* increased expression of disintegrin and metalloprotease with a thrombospondin repeat 1 (ADAMTS1) (Keightley et al., 2010b). Chemokines play an important role in normal uterine function; however, dysregulated chemokines can contribute to uncontrolled proliferation, migration and invasion and contribute to an inflammatory microenvironment (Kayisli et al., 2002). Chemokines CXCL8 and CXCL1 are both targets of PGF_2α_ (Sales et al., 2009; Wallace et al., 2009). CXCL8 leads to increased proliferation of Ishikawa cells *in vivo* (Sales et al., 2009) while CXCL1induces neutrophil chemotaxis *in vitro* and *in vivo* (Wallace et al., 2009). The effects of these chemokines presumably also involve activation of the PGF_2α_ receptor. A pro-angiogenic and anti-angiogenic relationship has been described as a result of PGF_2α_ and FP receptor interactions (Sales et al., 2005; Keightley et al., 2010b; Keightley et al., 2010a). PGF_2α_ and FP receptor activation enables endothelial cell network formation and proliferation *via* FGF2-FGFR1 signaling *in vitro* (Keightley et al., 2010a) and through transactivation of EGFR and secretion of VEGF leads to an increase in angiogenesis *in vivo* (Sales et al., 2005). Elevated COX-2 is associated with increased VEGF, PGE_2_ and angiogenesis in endometrial cancer and can be negatively regulated by miR-101 and while a specific receptor was not identified in this study it is feasible that FP and PGF_2α_ could play a role by conversion of PGE_2_ to PGF_2α_ (Dozier et al., 2008; Liu Y. et al., 2018).

### Cyclooxygenase in Uterine Tumor Microenvironment

The production of PGE_2_ by malignant endometrial epithelial cells in the tumor microenvironment can cause an increase in COX-2 expression. In normal endometrial cells, activation of transcription *via* NF-kB and stabilization of COX-2 mRNA leads to production of PGE_2_ (Tamura et al., 2002). PGE_2_ signals through four different E series receptors EP1-EP4 which are linked to different intracellular signaling pathways (**Figure 1**). The expression of the EP1 receptor was only increased in endometriotic tissue compared to healthy endometrium and tumor tissue; and in the tumor stroma, the expression of EP1 in the tumor was lower than in both normal tissue and endometriosis (Zhu et al., 2018). There was no significant differences in EP1 staining in either epithelium or in stroma within histological stage, grading, metastatic and recurrent subtypes (Zhu et al., 2018). High expression of EP3 was associated with impaired progression free survival and inhibition of EP3 signaling lead to a decrease in proliferation and migration in RL95-2 endometrial cancer cells (Zhu et al., 2017). Compared to EP1 and EP3, the EP2 and EP4 receptors are the most prominent receptors in regard to playing a role in uterine cancer pathogenesis. Jabbour et al. (2001) demonstrated an increase in expression of EP2 and EP4 receptors in endometrial adenocarcinomas and signaling through these receptors resulted in significantly higher cAMP production in response to PGE_2_. Signaling of the EP2 receptor in endometrial adenocarcimoma leads to transactivation of EGFR through PKA and c-Src and induction of VEGF (Sales et al., 2004b). Abera et al. (2010) reported on crosstalk between FP and EP2 receptors that results in increased cAMP release *via* the Gα_q_-Ca^2+^-calmodulin pathway. Endogenous PGE_2_ and exogenous PGE_2_, through exposure of seminal plasma, leads to expression of FGF2, a potent mitogenic and angiogenic factor, though activation of EP_2_ (Battersby et al., 2007). Exposure to PGE_2_ leads to an increase in phosphorylation of Akt and tuberin, a tumor suppressor that negatively regulates cellular proliferation, with a concomitant decrease in total tuberin protein expression (Sales et al., 2004a). In this study, the authors used inhibitors to block PI3K/Akt signaling and while involvement of a specific EP receptor was not identified (Sales et al., 2004a) several studies have determined that the EP4 receptor can lead to activation of PI3K pathway (Xu et al., 2018; Lu et al., 2019; Osawa et al., 2020).

Based on the studies presented in this section, there is a significant role for prostanoid signaling in uterine cancer development and progression. It would be interesting to see if other prostanoids as well as eicosanoids in LOX and P450 pathways also play a role in uterine cancer. It would also be beneficial to see additional studies combining inhibition of specific prostanoid receptors with first-line therapies.

## Eicosanoids in Cervical Cancer

Cervical cancer is one of the foremost contributors to worldwide gynecological malignancies and the top contributor to that in Southern Africa (Sales and Katz, 2012; Adefuye et al., 2014). In 2008, as reported by the International Agency for Research on Cancer, an estimated 493,243 women were diagnosed with cervical cancer annually, contributing to approximately 27,300 deaths (Sales and Katz, 2012). Cervical cancer primarily arises as a neoplasm that’s initiated by oncogenic variations of human papillomavirus (HPV) and of the 180 different genotypes of the infectious disease, 40 can be accredited to cause infection of the anogenital tract (Adefuye et al., 2014). The most common mode of transmission of HPV is through the exchanging of bodily fluids (primarily seminal fluid) during sexual intercourse (Adefuye et al., 2014). Not only does seminal fluid contribute to the transmission of HPV, but the presence of prominent concentrations of prostaglandins associated with seminal plasma (including PGE_2_) is likely to regulate inflammatory and tumorigenic pathways due to elevated expression of PGE_2_ receptors in cervical cancers (Adefuye et al., 2014). Increased binding of PGE_2_ to EP receptors can increase COX-2 expression as well as regulate target genes such as growth factors, angiogenic factors, prostaglandins, chemokines, and cytokines, which supports tumorigenesis, inflammation, and local changes in tissue architecture (Adefuye et al., 2014). Since COX-2 is controlled by growth factors, tumor promoters, oncogenes, and carcinogens, it is speculated that COX-2 expression is increased in a variety of cancers, including cervical (Saldivar et al., 2007). By producing prostaglandins, COX-2 is responsible for suppressing apoptosis and promoting tumor invasion (Saldivar et al., 2007). Colposcopically, within the same patient, COX-2 expression levels were measured at 83.1± 30.1 ng COX-2/mg protein in normal tissues versus 403.7± 175.6 in abnormal biopsies (CIN tissues). Inflammation is associated with carcinogenesis and in line with this association, Saldivar found that COX-2 levels were 3.7 times higher in specimen that were inflammation positive and of those specimens, COX-2 protein levels were 7.4 times higher than that in control biopsies (Saldivar et al., 2007). These data provide evidence that COX-2 may play an integral role in cervical cancer inflammation and tumorigenesis.

Upregulation of COX-2 can be attributed to HPV oncoproteins playing a role in cervical carcinogenesis. Kim and colleagues investigated the role of HPV 16, the most frequent genotype detected in cervical cancer tissue, E5 oncoprotein in cervical cancer development (Kim et al., 2009b). One-way E5 affects COX-2 levels is through EGFR activation; in HaCa T cells transfected with E5, there is activation of the EGFR pathway (Kim et al., 2009b). Transactivation of EGFR by protease-activated receptors are also known to have an effect on COX-2 upregulation (Almeida et al., 2018). Inhibition of EGFR activation lead to a decrease in COX-2 expression (Kim et al., 2009b). E5 also induces COX-2 mRNA expression; assessment of transcriptional activity determined that the COX-2 gene expression was regulated through binding of NF-κB and AP-1 to the COX-2 promoter (Kim et al., 2009b). Inhibition of NF- κB or AP-1 lead to significant decrease in COX-2 expression (Kim et al., 2009b). Bcl proteins are essential for cell clearance and when defective, can promote cancer (Alibek et al., 2014).

Sales and Katz (2012) have shown that PGE_2_ biosynthesized by COX-1 and COX-2 in HeLa cells, increases expression of pro-angiogenic factors that can exert paracrine activity on endothelial cells to promote blood supply for tumor growth and alter vascular permeability for the release and distribution of leucocytes and macrophages to surrounding tissues. The production of prostaglandins *via* elevated COX enzyme activity, act on G-protein couple receptors (GPCRs) to encourage tissue remodeling for tumors, angiogenesis, apoptosis inhibition, cell proliferation, and altered vascular permeability (Adefuye et al., 2014). Peng et al. (2019) examines anti-cancer activity of medicinal plant, *Conyza blinii*, through the downregulation of COX-2 and decrease in PGE_2_ levels. *C. blinii* acts as a NF-κB pathway inhibitor and it inhibits downstream gene expression of COX-2, which in turn decreases PGE_2_ production (Peng et al., 2019). In *C. blinii* treated mice, PGE_2_ levels in both serum and tumors were significantly lower. This decrease in prostaglandin activity has promising implication in inflammation and immunomodulation (Peng et al., 2019).

The Bcl family is important in regulating apoptosis according to environmental cues. Bcl-2 homologues act as oncoproteins that are anti-apoptotic and thus, integral for cancer initiation (Alibek et al., 2014). Viruses have been known to have developed a mechanism of apoptosis prevention in host cells through targeting Bcl-2 homologues. HPV protein E6 interacts with p53 in infected cells in order to prevent p53 from blocking the anti-apoptotic Bcl-2 protein (Alibek et al., 2014). It is unknown how viral protein E5 interacts with the Bcl family directly; however, it is known that as pro-apoptotic Bak and Bax proteins are decreased, Bcl-2 expression levels are increased thus inhibition of Bak and Bax is facilitated by E5 (Alibek et al., 2014). This inhibition is performed through ubiquitin-proteasome-dependent degradation, involving EGFR activation and subsequently COX-2 upregulation (Alibek et al., 2014).

In addition to the contribution of HPV in cervical cancer, HIV also poses a role in infection and inflammation through elevation of COX-2 expression as well as elevated PGE_2_ levels Fitzgerald and colleagues (Fitzgerald et al., 2012). HIV infected women are five times more likely to develop cervical cancer than HIV-negative women. HIV induces high COX-2 levels in a number of immune cells circulating and in tissue (Fitzgerald et al., 2012). Fitzgerald and colleagues hypothesized that HIV promotes COX-2 levels in cervical tissue and in turn increases systemic levels of PGE_2_ leading to inflammation and cancer (Fitzgerald et al., 2012). Women positive for HIV and negative HPV have significantly higher levels of COX-2 mRNA (+/−) (*P*<0.001) with additional increase in COX-2 observed in women positive for both HIV and HPV (+/+)(*P*<0.001). Similarly, urinary PGE-M levels were elevated in HIV positive women; HPV positive women did not exhibit significantly higher levels of PGE-M meaning HIV status was the only statistical predictor of PGE-M levels. Through this observation, it was concluded that COX-2 and PGE-M levels were positively correlated (*P*= 0.005) (Fitzgerald et al., 2012). This study confirms association of HIV with development of cervical cancer. The data presented in this section demonstrates a significant role for COX-2 and PGE_2_ in the pathogenesis of cervical cancer alone and in conjunction with infection by HPV or HIV.

## Eicosanoids in Vulvar Cancer

Vulvar cancers are rare, accounting for only an estimated 6,070 cases and 1,280 deaths in the United States (US) in 2019 (Siegel et al., 2019). The majority are squamous cell carcinomas (Moroney et al., 2013). Vulvar cancer arises from two pathways (Zarcone et al., 1997). The most common mechanism involves chronic inflammation in older women from vulvar dystrophies such as lichen sclerosus with development of differentiated vulvar intraepithelial neoplasia (VIN) then keratinizing squamous cell carcinoma. In 25–30% of cases and often in younger women, pathogenesis is driven by human papillomavirus (HPV) leading to usual VIN or high-grade squamous intraepithelial neoplasia (HSIL) and basaloid or warty squamous cell carcinoma. HPV-related vulvar cancers may exhibit a predilection for localization to the perineum and carry a favorable prognosis (Hinten et al., 2018) compared to vulvar cancers that arise independent of HPV.

COX-2 expression has clinicopathologic relevance in vulvar cancer. Lee et al. (2007) found that COX-2 expression by immunohistochemistry and adjacent inflammatory cell infiltrate was higher in older compared to younger vulvar cancer patients (P = 0.002) (Lee et al., 2007). Interestingly, COX-2 expression was inversely correlated with differentiation. Fons et al. (2007) found that strong COX-2 expression was an independent predictor of poor disease-specific survival (HR 4.01, 95% CI 1.10-14.63, P = 0.035), as was lymphovascular space invasion, lymph node involvement, tumor size > 4 cm, and absence of apoptotic caspase-3.

Inhibition of COX-2 may potentiate the effects of chemotherapies used to treat vulvar cancer. Dual treatment of COX-2-expressing vulvar squamous cell carcinoma cell lines (A431 and SW962) with the COX-2 inhibitor celecoxib and cisplatin inhibited 49% of growth, compared to 25% with cisplatin monotherapy alone after 48 h (Kim et al., 2009a). Interestingly, exposure of the cells to either agent at 10 micromol/L or in combination increased COX-2 expression, putatively through downregulation of its negative regulator PI3K. Inhibition of PI3K has been shown to block activation of mitogen-activated kinase (MAPK) independent of EGFR in A431 cells (Graness et al., 2000), suggesting a prominent role for therapies targeting PI3K in vulvar cancer and as mentioned previously signaling through PGE_2_ receptor EP4 can lead to activation of PI3K. In squamous cell carcinoma of the head and neck, agonists of the PGE_2_ receptor EP4 promote migration through PI3K activation and Ca^2+^ influx (Osawa et al., 2020). EP4 antagonists are currently under study in phase IB trials of rectal cancer in conjunction with pre-operative chemoradiation (Adlai Nortye Biopharma CO, Ltd). Based on molecular mechanisms, it may be hypothesized that EP4 inhibition may one day merit study in vulvar cancers and other gynecologic malignancies treated with chemoradiation.

## Eicosanoids in Vaginal Cancer

Vaginal cancer represents only 1–2% of gynecologic malignancies and is under-studied due to its rarity. There were only 5,350 US cases and 1,430 deaths from this disease in 2019 (Siegel et al., 2019). Approximately 10–50% of patients with vaginal cancer or its precursor lesion [high grade vaginal intraepithelial neoplasia (VAIN)] have a history of hysterectomy or radiation for cervical cancer. HPV infection and immunosuppression are strong risk factors for development. The majority arise in the upper third of the canal and involve the posterior wall and are squamous in histology. Less common subtypes include clear cell adenocarcinoma, which is characteristically associated with *in utero* diethylstilbestrol (DES) exposure or malignant degeneration of endometriosis, and embryonal rhabdomyosarcoma, the most common vaginal malignancy in children (Cardenes et al., 2013).

COX-2 expression has been found to be a prominent feature in mucosal inflammation of the vagina leading to disease susceptibility and transmission (Joseph et al., 2012), as well as rectovaginal endometriotic implants (Fagotti et al., 2004). Recently, we have observed lower expression of EP4 in vaginal tissues of patients with pelvic organ prolapse and higher expression in women with radiation-induced vaginal stenosis after treatment for gynecologic malignancy compared to healthy controls (Santayana et al., 2018). There also appears to be differential expression between luminal, basal, and intermediate (smooth muscle) layers of the vagina. Additional studies are underway to better elucidate the role of EP4 in both alterations of tissue integrity as well as its relevance in gynecologic malignancy, but this preliminary data raises interest in a possible pharmacologic role for use of EP4 inhibitors to modify a number of biologic processes in vaginal cancer.

## Conclusion

This manuscript aimed to provide a comprehensive review of the role of eicosanoids in gynecological malignancies. With increasing evidence, it is demonstrated that eicosanoid driven processes are associated with the progression and spread of gynecological malignancies. These bio-active lipids have direct effects on cancer cells as well as indirect effects on the tumor microenvironment. Most of the studies reported in gynecological malignancies focused on the cyclooxygenase pathway especially the role of PGE_2_ and PGF_2α_ and their receptors. The least reported pathway was the P450 cytochrome epoxygenase pathway in which only ovarian cancer had associative studies involving the pathway but with a lack of mechanistic investigation; thus, this pathway is one that should be examined more in the future for all gynecological malignancies.

Given the rare nature of vaginal and vulvar cancer, there is not an abundance of research available; however, the studies performed are directed towards the cyclooxygenase pathway and exploring all of the eicosanoid pathways would be of benefit in order to find much needed treatments and information related to tumorigenesis of these malignancies. It was demonstrated that the COX pathway plays a significant role in the pathogenesis of cervical cancer alone and in conjunction with HPV and HIV; however, there was a lack of mechanistic studies in interrogating which receptors are involved in the tumorigenic signaling pathways. In uterine cancer, it would be of benefit to see more studies performed on the potential for PGE_2_ receptors EP1 and EP3 in uterine cancer given that to date most of the investigations have been correlative.

In ovarian cancer, the data presented for the potential role for the lipoxygenase pathway in ovarian cancer tumorigenesis and modulation of the tumor microenvironment supports the need for additional studies including antagonizing the receptors for both leukotrienes and HETEs. Given the potential anti-neoplastic effects on some of the prostaglandins such as the cyclopentenone prostaglandins as well as the association of PGD_2_ with good prognosis in high grade serous ovarian cancer (Alves et al., 2019) as well as the positive effects of blocking PGE_2_ EP4 in modulating the tumor microenvironment (Hennequart et al., 2017), additional mechanistic studies exploring modulation of COX signaling downstream of the COX enzymes as a monotherapy and in combination with additional therapeutic interventions is warranted.

Overall there is a theme that inhibition of COX pathways leads to potentiation of chemotherapeutic treatment in multiple gynecological malignancies including ovarian (Li et al., 2012a; Li et al., 2013), uterine (Hasegawa et al., 2013; Reader et al., 2019) and vulvar (Kim et al., 2009a). Additional studies are warranted to explore the role of and the mechanism behind adding inhibition of prostanoid receptors to chemotherapy and other treatment modalities in order to conserve potential anti-neoplastic eicosanoid activities for development of novel treatments for gynecologic malignancies.

## Author Contributions

PS, DR, GR, and JR contributed written sections and figures. MC and AF contributed edits. All authors contributed to the article and approved the submitted version.

## Funding

The work presented has been supported by Department of Obstetrics, Gynecology, and Reproductive Medicine (JR, GR, DR), the Kaleidoscope of Hope Ovarian Cancer Foundation (JR), The Foundation for Women’s Cancer Research (DR), Maryland Department of Health’s Cigarette Restitution Fund Program (AF, JR), Baltimore Veterans Affairs Administration (AF) the STAR-PREP program of the National Institute of General Medical Sciences at the University of Maryland School of Medicine (MMC).

## Conflict of Interest

The authors declare that the research was conducted in the absence of any commercial or financial relationships that could be construed as a potential conflict of interest.
